# Statistical models discriminating between complex samples measured with microfluidic receptor-cell arrays

**DOI:** 10.1371/journal.pone.0214878

**Published:** 2019-04-08

**Authors:** Ron Wehrens, Margriet Roelse, Maurice Henquet, Marco van Lenthe, Paul W. Goedhart, Maarten A. Jongsma

**Affiliations:** 1 Biometris, Wageningen University & Research, Wageningen, The Netherlands; 2 Bioscience, Wageningen University & Research, Wageningen, The Netherlands; 3 Laboratory of Plant Physiology, Wageningen University & Research, Wageningen, The Netherlands; University of Illinois at Chicago, UNITED STATES

## Abstract

Data analysis for flow-based in-vitro receptomics array, like a tongue-on-a-chip, is complicated by the relatively large variability within and between arrays, transfected DNA types, spots, and cells within spots. Simply averaging responses of spots of the same type would lead to high variances and low statistical power. This paper presents an approach based on linear mixed models, allowing a quantitative and robust comparison of complex samples and indicating which receptors are responsible for any differences. These models are easily extended to take into account additional effects such as the build-up of cell stress and to combine data from replicated experiments. The increased analytical power this brings to receptomics research is discussed.

## Introduction

Receptomics research with microfluidic receptor cell arrays aims to measure purely biological responses without a complicated biological system surrounding it [1–3]. For example, the human tongue can be emulated on a chip by an array containing G-protein coupled receptors (GPCRs), *e.g*., in the form of reconstituted receptor proteins [4] or vesicles [5]. Another possibility is formed by living cells expressing the genes coding for particular GPCRs, produced by either reverse-transfecting a generic cell line on the chip [6] or spotting pre-transfected cells [7]. Such a tongue-on-a-chip allows direct access to the original taste signal, before the signal is further transmitted via neurons, and processed and interpreted by the human brain. Thus, while a taste panellist would define a sample as bitter or sweet, a tongue-on-a-chip provides direct quantitative information on which taste receptors are triggered, and by how much. On a more general level, receptomics enables identifying compounds or extracts activating or blocking specific receptors active in taste sensation as well as in many other processes. Humans have a wide palette of receptor proteins. Even considering only GPCRs there are more than 800 receptors for the detection of hormones, neurotransmitters, tastants, odorants, and others. Since all receptors play an important role in human physiology, there is an advantage to a receptomics approach aiming at combining different receptors on a single chip, allowing the researcher to study the role of a compound or extract in a wider perspective by including all or at least the most relevant receptors.

However, data analysis for flow-based in-vitro biosensor arrays such as a tongue-on-a-chip is complicated by the relatively large variability in specific and non-specific responses within and between arrays caused by differences in the expression of the transfected receptor DNA, variability in spots and in cells within spots. Approaches based on simply averaging the response values of spots of the same type therefore lead to estimates with a large variability, allowing only the most obvious differences between samples to be detected and providing no means to correct for other effects often seen in flow-cell based approaches such as time or memory effects. Furthermore, chemically complex samples may trigger host-cell responses that vary widely between receptor types, depending on unknown interactions with the functional properties of the transfected DNA [8]. To eliminate such host-cell responses often hampering the analysis, one may dilute the sample until the host-cell response is no longer observed, but that often also means losing a large part or even all of the signal.

Here, we are focusing on microfluidic receptomics chips containing receptor cell arrays generated by reverse transfection of DNA arrays and with ectopic expression of different GPCRs and a generic calcium-ion sensor protein, Twitch2B [6, 9]. The chips were printed with plasmid DNA encoding a GPCR gene and a calcium sensor gene. Transient expression of the genes was achieved by reverse transfection: HEK293 cell were seeded on top of the DNA array, the DNA was taken up and the genes were expressed leading to G-protein-coupled receptors embedded in the cell membrane and calcium sensor accumulation in the cytoplasm. Upon stimulation of the receptor with a ligand, the GPCR signal transduction pathway is activated via G*α*16GUST44 [10]. Activation of this chimaeric G-protein leads to a transient rise in calcium ion concentration within the cytoplasm. These calcium dynamics can be measured using ratiometric calcium sensors that are based on the FRET pair CFP and YFP connected by a calcium binding domain.

**Fig 1:**
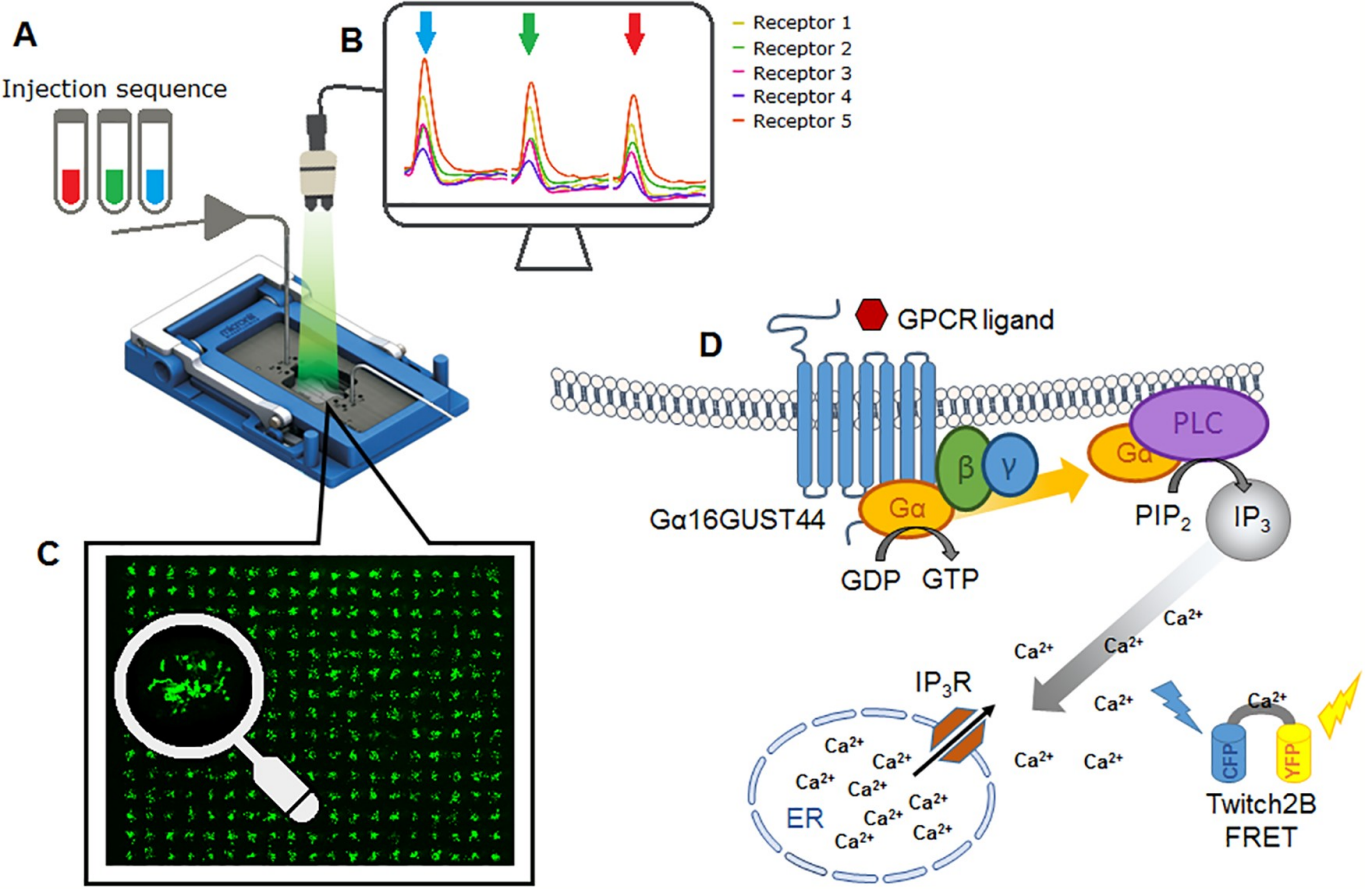
The receptomics principle used in this paper. The microfluidic system (A) allows sequential injections of samples into the flowcell. The fluorescence microscope in (B) captures CFP- and YFP-FRET images of the entire cell array that can be analysed to give average values for the YFP/CFP fluorescence ratio. (C) The spots of the array are composed of approximately 50 fluorescent cells, each transfected with a receptor-coding gene and a fluorescent calcium sensor coding gene. Both receptor and sensor are expressed in each fluorescent cell of an array spot (D). Each spot on the array expresses a different receptor type. When the receptor interacts with a ligand, the G*α*16GUST44 signalling cascade is activated: the G*α* protein acts via Phospholipase C (PLC) to convert PIP_2_ into second messenger IP_3_ which can interact with IP_3_ receptors (IP_3_R) on the Endoplasmic Reticulum (ER). The IP_3_R are calcium ion channels which, when activated, transiently release calcium into the cytoplasm. The Twitch2B calcium sensor, expressed in the cytoplasm, is a fluorescent FRET probe which, upon binding of calcium ions, changes conformation and thereby increase the FRET efficiency between the CFP and YFP fluorescent proteins. These FRET changes are captured by the camera as a ratio change of CFP and YFP intensities.

The principle of the sensor is depicted in Fig 1. Since the array can contain hundreds of spots, it is possible to accomodate many different receptors and at the same time have a relatively large number of spots for each receptor type, leading to more precise estimates. For each spot on the array, fluorescence time series are measured at two different wavelenghts which are, in a series of steps, transformed in response values for each spot upon exposure to a sample [6]. The goal of the statistical analysis, the main focus of this paper, is to be able to unambiguously and objectively discriminate between samples in terms of receptors affected.

## Materials and methods

### Cell array experiments

Reverse-transfected cell arrays were prepared and measured as previously described in [6]. The genes encoding bitter receptors were obtained from genomic DNA by PCR amplification and cloned into pcDNA3 containing the N-terminal sstr3 tag (gift from Dr. Wolfgang Meyerhof, German Institute of Human Nutrition Potsdam-Rehbrücke, Germany). Plasmid pcDNA3 Twitch2B (addgene #49531 [9]) was added to the print solution to enable ratiometric calcium detection. The arrays contained 24 individual bitter taste receptors, including SNP variants Tas2R4-SLN and -FVS, Tas2R38-PAV and -AVI, and Tas2R39-A and -T. For a complete overview, see—in the remainder of this paper, the Tas2 prefix will be deleted for clarity. These receptors were placed randomly on the array, to avoid location and neighbour effects. Each print mix contained 75ng/*μ*l receptor-coding plasmid DNA and 10 ng/*μ*l calcium-sensor-coding plasmid DNA. The low level of sensor expression obtained this way prevented buffering of the calcium ions by the sensor itself. Two additional controls were printed; one control without receptor-coding DNA but an empty vector instead (Mock), and one control without receptor-coding DNA but with a modified calcium sensor protein that lacks the ability to bind calcium. This control, named YC-, has a YFP/CFP ratio independent of the intracellular calcium concentration. Any environmental influences on the spectral properties of the fluorescent calcium probe will be detected by this control, as will be fluorescence coming from the sample itself.

Reverse-transfected cell arrays were prepared using HEK293 cells stably transfected with G*α*16GUST44 (a gift from Dr. Takashi Ueda, Nagoya City University, Nagoya, Japan). At 48 hours after transfection, the cell arrays were removed from the incubator, washed and incubated in assay buffer for 1 hour prior to the measurements. All measurement series were performed using a 150 *μ*l flowcell and the flowcell holder (Micronit Microfluidics B.V., Fluidic Connect PRO Chip Holder.) The assay buffer (NaCl 130mM, KCl 5mM, Glucose 10mM, CaCl2 2mM, HEPES 10mM at pH 7.4) was set to a continuous flow of 300 *μ*l/min over the array. The injections were performed with a manual valve containing a 150 *μ*l sample loop. Reagents used in the injections were Adenosine 5’-triphosphate (ATP, Sigma A6419), Chloramphenicol (Duchefa C0113.0100), Picrotoxinin (Sigma P8390), 6-propyl-2-thiouracil PROP (Sigma P3755) and D-Salicin (Wacko 199-00083). The time between injections was set to approximately 5 minutes, allowing the intracellular calcium levels in the solution to return to pre-injection values.

### Image analysis

Upon stimulus by their respective ligands, receptors will induce a signal transduction leading to an increased calcium ion concentration in the cytosol. This can be monitored in real time by means of FRET (Förster Resonance Energy Transfer) imaging [6], here using a Leica fluorescent stereo microscope (Leica M205FA with DFC 345 FX camera). Two channels were monitored, CFP (ET CFP 10447409, excitation 436/20 and emission 480/40) and YFP (ET FRET 10450566, excitation 436/20 and emission 535/30), respectively. Note that CFP and YFP images are taken alternatingly and therefore have different time points. The CellProfiler software package [11] was used to separate signal pixels from the background to define a grid corresponding with the positions of the spots and to quantify the raw CFP and YFP signals.

### Preprocessing

Processing the images leads to a data table containing, for each of the spots, the CFP and YFP signals for each measurement cycle. As a first preprocessing step, the data are corrected for fluctuations in the lamp output using a reference position outside the flowcell. Next, spots containing fewer than fifteen pixels are removed from the data. Receptor types that are represented by fewer than five spots are removed altogether. The CFP and YFP signals are then smoothed using cubic smoothing splines [12]. The *signal* of each spot, related to the calcium concentration in the cell, is now defined as the ratio of the interpolated CFP and YFP signals at specific time points. An example showing CFP and YFP data (after lamp correction) for one spot, as well as the derived spot signal, is shown in S1 Fig.

Next, this spot signal is used to calculate the response of a spot to a sample injection. This can be done in several different ways: since spot signals often show peak-like shapes, obvious candidates are peak height and peak area. Here, we focus on the the increase or decrease of a spot signal after the injection, given by the ratio of the extreme value of the signal (within a certain time window), and the average of the first three signal values directly after the injection. In this case, the time window is chosen to cover 30 cycles (approx. 1,5 minutes) directly after the sample introduction, corresponding to the time the cells are exposed to the sample. An example is provided by S2 Fig. In this way, a data matrix is obtained that describes the quantitative response of each spot to each sample injection, independent of the initial signal strength.

### Statistical modelling

#### Qualitative analysis

To obtain an easily interpretable overview of the differences between samples for which multivariate responses are available, Principal Component Analysis (PCA [13, 14]) is often used. In PCA, a high-dimensional data matrix is reduced to a much lower number of dimensions (for visualization purposes usually two) that contains the maximum amount of information. Each new dimension is a linear combination of the original variables. So-called score plots show the position of the samples in this reduced space; loading plots show the weights of the original variable on the new axes, *i.e*., which of the original variables are important in each new direction. Quantitative statements about significant differences between injections cannot be made with PCA. In a context where samples are compared to see whether they lead to different taste receptor responses PCA therefore is of limited value, and a statistical model is needed.

#### Mixed models

Here, we are interested in assessing which samples differ *significantly* from each other, and which receptor types are responsible for any observed differences. Our approach is based on linear mixed models [15]. In the simplest form, one fits a model describing the spot response *R* with receptor and sample as (fixed) variables, also including the interaction between the two. Additional fixed variables could include the injection number, to account for any trends in sensitivity over time, and array number, when combining several replicated experiments in one analysis. To take account of the fact that every spot will have its own characteristics, the spots have to be part of the model, too. In order to avoid estimation of individual coefficients for all spots, which would consume valuable degrees of freedom, one can include spots as a so-called random variable. That is, one assumes that the spot effects follow a normal distribution around zero, and the only parameter that is estimated is the width of the distribution, implying that observations on the same spot are correlated rather than independent. This leads to the following model (in matrix notation):

y=Xβ+Zu+ε

where *y* is the vector of spot responses to sample injections, *β* contains the coefficients for all fixed variables, and *u* contains the random effects associated with the individual spots. *X* and *Z* are design matrices relating the responses to the values of the independent variables: *i.e*. *X* contains information about the sample type, receptor type and possibly injection number and array number, and *Z* describes the receptor types present at all spots. Finally, *ε* is the vector of residuals. The model is fit, as is common practice, through restricted maximum likelihood (REML) [15].

#### Contrasts

Once the model is obtained, it can be used to estimate the expected response of a particular type of receptor to a particular sample (so-called estimated marginal means). By focusing on differences of these estimates for individual spots, the differences between the spots are eliminated, removing a major source of irrelevant variation and leading to much narrower confidence intervals and increased statistical power. Such differences are called *contrasts*, and can be defined in a number of ways. In *treatment-versus-control contrasts*, for example, one of the injection types is used as a control, and the magnitude of the results of the other injection types (usually the study samples) is related to this. In this way one can quantitatively assess which receptors show different responses to the injection of different samples.

#### Scaling of the response variable

Although the mixed models can be fitted for the intensity ratio described above and shown in S2 Fig, it is more appropriate to use a log-scaled intensity ratio as the response variable. Comparing samples then will (after backtransformation) lead to ratios of ratios: a value of 1 corresponds to no change in intensity ratio, values lower than 1 correspond to a decrease in intensity ratio, and values higher than 1 to an increase. In this way a treatment-versus-control contrast can be expressed in terms of simple ratios of the original CFP and YFP responses, which would not have been possible if we would have analysed the intensity ratio data without the log transformation. The variance-stabilizing effect of the log transform is less important here since all intensity ratios are relatively close to one; for other response variables with heteroscedastic residual variance, this could be an important reason to employ the log transform.

### Software

All statistical analyses described in this paper have been performed using R [16], using packages **nlme** [17] for fitting the mixed models, **emmeans** [18] for obtaining estimated marginal means, contrast estimates and confidence intervals, and **lattice** [19] for generating plots. R scripts and data to reproduce the results in this paper are available in S1 Code, S1 and S2 Data, respectively.

A stable version of the analysis scripts using defaults also used in this paper has been included in our “Receptomics” software, which provides an intuitive and powerful user interface allowing inspection of raw data, elimination of bad data points, choice of model (“raw” effect sizes or treatment-versus-control contrasts), and inspection of the outcome of the statistical modelling. For more information about this software, see http://www.receptomics.com.

## Data

Three experiments, each executed three times, serve to illustrate our approach. In the first type of experiment (A), a quality control (QC) mixture of four compounds, chosen because they are known to hit specific bitter receptors, was injected at nine different dilutions (see Table 1). The sample with the lowest concentration was injected first; each subsequent injection had a double concentration of the QC mixture. In addition, a blank injection, and a 2 *μ*M ATP injection were performed. The ATP injection elicits a host-cell response, different for each receptor type. The second type of experiment (B) was set up to compare 2 *μ*M ATP injections with injections where the ATP sample was spiked with the same QC mixture as in (A). Also here a blank injection (containing only assay buffer) was included. This experiment is a simple example of a case where a specific response should be estimated in the presence of a constant background (the host-cell response). Table 2 gives an overview of the injections in both experiment types.

**Table 1:** QC mixture and target receptors, in order of expected sensitivity [20]. Concentrations of compounds in the A-type experiments correspond to the most concentrated sample. The concentrations of the QC mixture in the B experiments correspond for all compounds but D-Salicin to QC 2 in the A experiments.

Spike	Exp A (*μ*M)	Exp B (*μ*M)	Affected receptors
Chloramphenicol	500	250	R8 > R46 > R1, R10, R43 > R39
Picrotoxinin	500	250	R14 > R46 > R1, R10
D-Salicin	10,000	1,000	R16
PROP	20	10	R38 PAV

**Table 2:** Injections for experiments A and B. The numbers after “QC” in Experiment (A) indicate the dilution factors: samples have been injected in order of increasing concentration.

Injection	Experiment (A)	Experiment (B)
1	Blank	Blank
2	QC 256	QC
3	QC 128	ATP 2uM
4	QC 64	ATP 2uM+QC
5	QC 32	ATP 2uM
6	QC 16	ATP 2uM+QC
7	QC 8	ATP 2uM
8	QC 4	ATP 2uM+QC
9	QC 2	ATP 2uM
10	QC 1	QC
11	ATP 2uM	

## Results

### Experiment A

**Fig 2:**
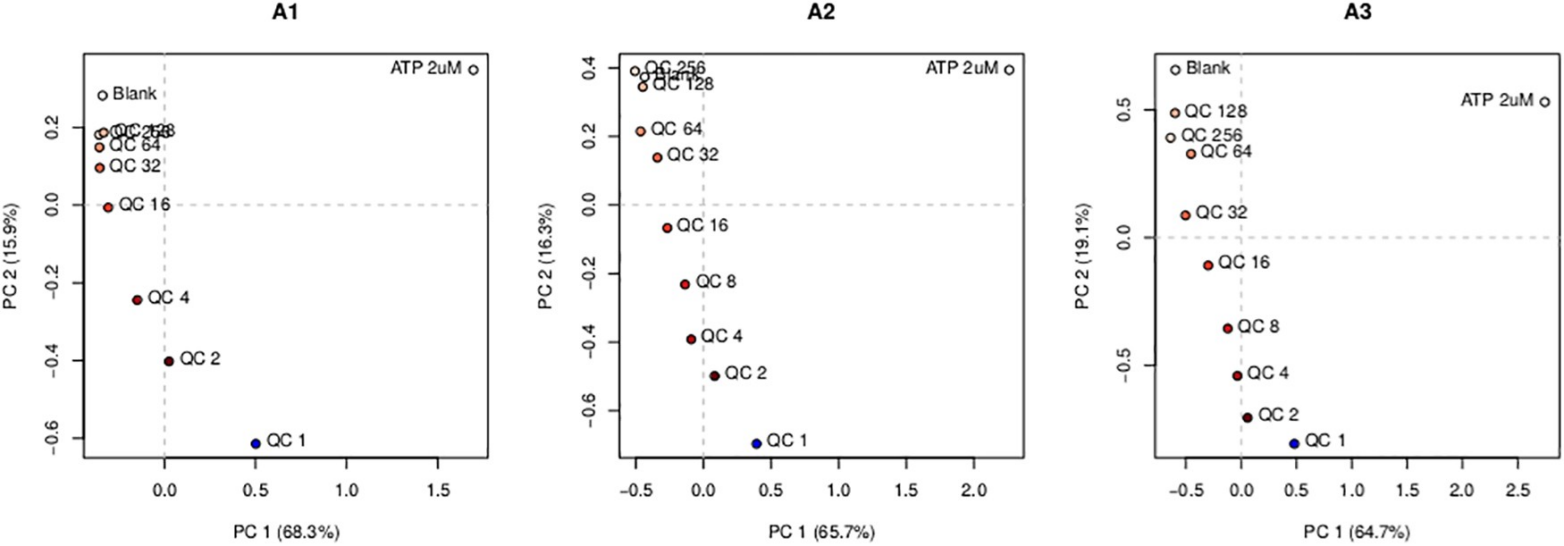
PCA score plots for the three replicated experiments, type A. Dots correspond to injections. In all three cases, the ATP injections lie in the top right corner, and the blank injections in the top left corner. Injections of the QC mixture move away from the blank injection with increasing concentrations.

Experiment type A investigates spot responses to exposure to a dilution series, and was executed three times, using three identical arrays. The QC 8 injection in the first replication (experiment A1) did not succeed, so data for that particular dilution are missing. The PCA score plots for the three replicated experiments are shown in Fig 2. One can clearly see the trend of more concentrated QC injections going away from the blank injection, while the ATP injection is in a completely different part of the PCA space. The corresponding loading plots, shown in S3 Fig, indicate which variables are involved in the first two PCA dimensions. Although some of the receptors, *e.g*., R8 and R14, seem to be important in PC2, the direction of increasing QC concentrations, the loading plots are hard to interpret because of the large number of variables and the fairly large variation, also within spots of the same receptor type.

**Fig 3:**
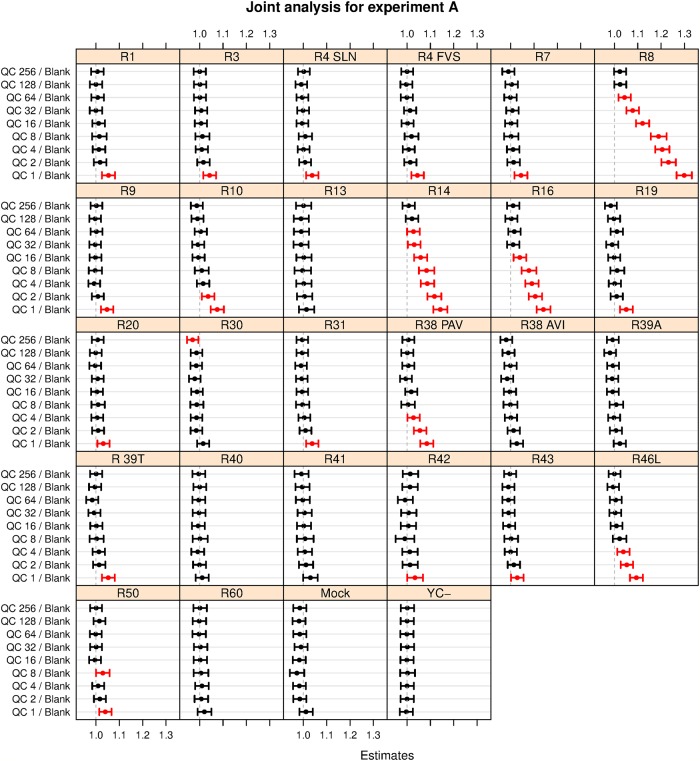
Control-versus-treatment contrasts from the joint analysis of the three type-A experiments. Each panel shows the estimated contrasts and associated 95% confidence intervals of a particular receptor. Contrasts are the ratio between the model response for a particular injection, and the reference injection, here the blank. Only the injections of the QC mixture are shown here to stress the pattern of increasing response with increasing concentration (from top to bottom, corresponding to decreasing dilutions indicated with the numbers on the y axis). Significant results, not including the baseline value of 1.0 in the confidence interval, are shown in red. For several receptors, e.g., R8 and R14, we see a clear relation between response and increasing concentration.

More quantitative results are obtained with the mixed-models approach, presenting for each receptor type an estimate of the response to the different injections, including standard deviations and confidence intervals. By considering the treatment-versus-control contrasts, variability between spots is eliminated. These contrasts, estimated from the model combining the three arrays in one single analysis, are shown in Fig 3. The Blank injection is used as the control. In this figure, significant effects, not including the value of 1 in the confidence interval, are shown in red. In total, 42 significant effects are found. Going from top to bottom in each panel, concentrations of the QC mixture increase (*i.e*., dilution factors become lower) leading to larger responses for several of the receptors. Although the individual arrays show some small differences, the general patterns are very similar. Very clear responses are seen for receptors R8, R14, and R16, at at somewhat higher concentrations also for R10, R38 PAV and R46L. All of these receptors are present in Table 1; receptors R1 and R39, also mentioned in the table, only show a significant response at the highest concentration. Note that some other receptors not present in Table 1 seem to respond, too—this may be a genuine response, since not for all receptors it is fully known what triggers them. The full results of the analysis of the individual arrays can be found in the supplementary material, S4–S6 Figs.

### Experiment B

In Experiment B the goal is to distinguish between two types of injections, both containing a large host-cell response. This host cell response, independent of the transfected receptor, is set up by means of a sample containing ATP. One type of injection consists of the ATP sample; the other adds to this the QC mixture also employed in Experiment A, so that both a generic host-cell response and specific receptor-specific responses are elicited. The statistical model then should indicate which receptors show significant differences between the two types of injections.

**Fig 4:**
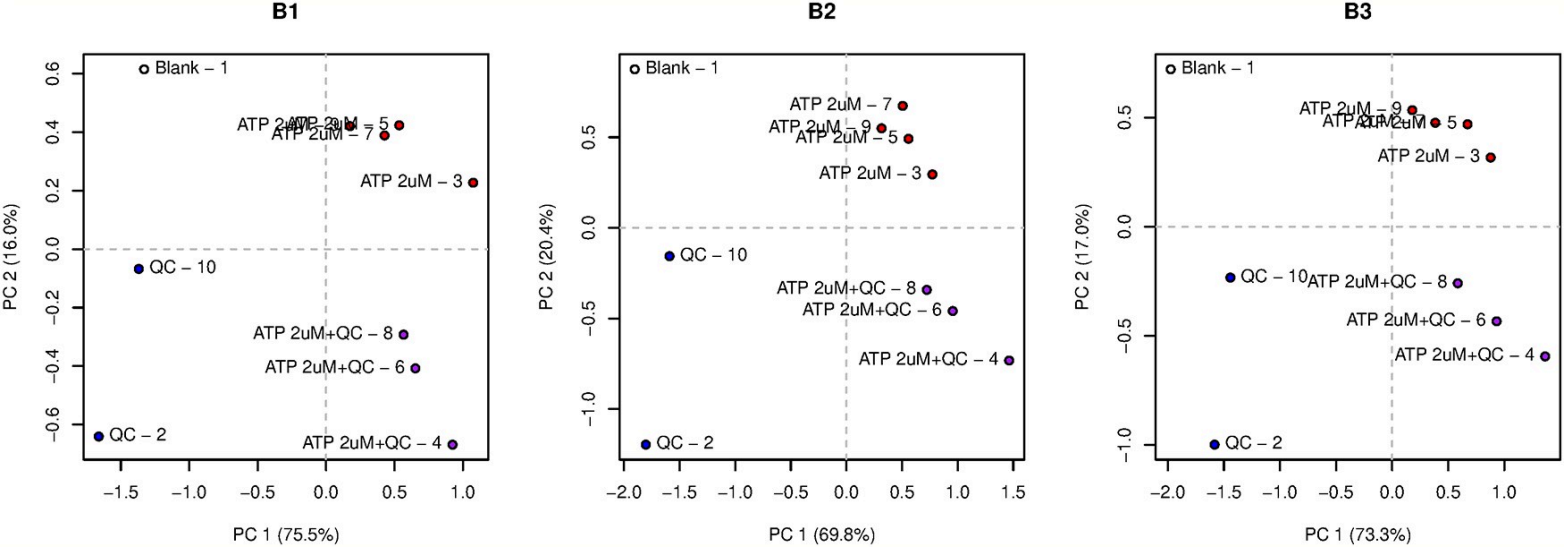
PCA score plots for the three replicated experiments, type B. Dots correspond to injections—the name of the injection type is followed by the injection number. In all three cases, injections without ATP are on the negative side on PC 1; injections without QCs are on the positive side on PC 2. There is a clear trend where later injections are located more towards the top left corner of the plot.

The PCA score plots are shown in Fig 4. In all three replicated experiments, the four injection types (Blank, QC only, ATP only, and ATP plus QC) are located in four different quadrants. Clearly, it is possible to distinguish between the injection types: the first PC distinguishes on the basis of the presence or absence of ATP; the second PC does the same for the QC mixture. However, when looking at the loading plots (see S7 Fig) it is not at all clear which receptors are involved. The two most responsive receptors to the QC mixture, R8 and R14, are the ones with the biggest loadings on PC2, but we see appreciable differences, also within one type of receptor.

To be able to concentrate on the most relevant results only, the linear mixed models are constructed using only the ATP and ATP+QC injections, leaving out the Blank and QC injections. Peak number is included as a (numerical) variable, allowing a trend over time. This is necessary: in the score plots of Fig 4 the effect of the injection order is clearly visible. Later injections of the same type tend to have higher values on the PC2 axis.

**Fig 5:**
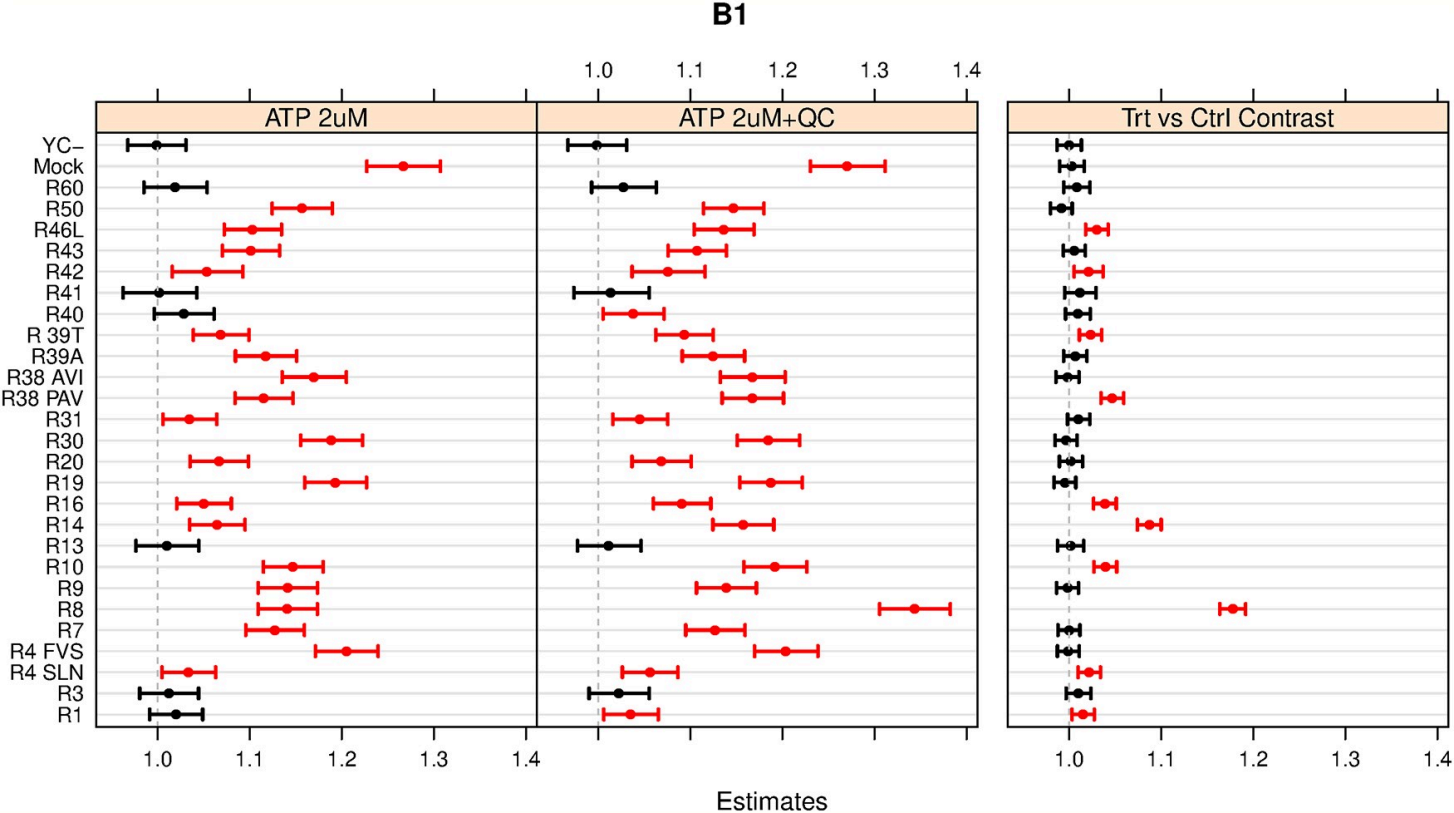
Estimates and 95% confidence intervals for the first B-type experiment. The panels on the left show the estimated responses and associated confidence intervals of all receptors to injection types. The right panel shows the corresponding spot-wise ratios, the treatment-versus-control contrasts. Significant results, not including 1.0 in the confidence interval, are indicated in red. Note that the confidence intervals in the right panel are much more narrow than those in the left two panels since the between-spot variation is taken out of the equation.

The results for the first of the three experiments, B1, are shown in Fig 5. The two left panels show the estimated response values, basically averages of model predictions for the two different injection types, for all receptors. It is remarkable to see how the responses to the ATP injections vary widely between host cells carrying different bitter receptors. Perhaps even more surprising is the fact that the response of the Mock receptor to ATP is the strongest of all. The right panel in Fig 5 shows the treatment-versus-control contrast calculated by the mixed model, where the ATP 2uM injection is the reference. Since the comparisons are effectively made *within* each spot, the confidence intervals for the contrasts are much more narrow than the confidence intervals for the estimated responses, leading to increased statistical power. In total, 10 significant treatment-versus-control contrasts, not including the value of 1 in the confidence interval, are found here. Note that for both control receptors, Mock and YC-, the contrasts are not significant, indicating that the model has effectively eliminated the very large host-cell response component to ATP.

**Fig 6:**
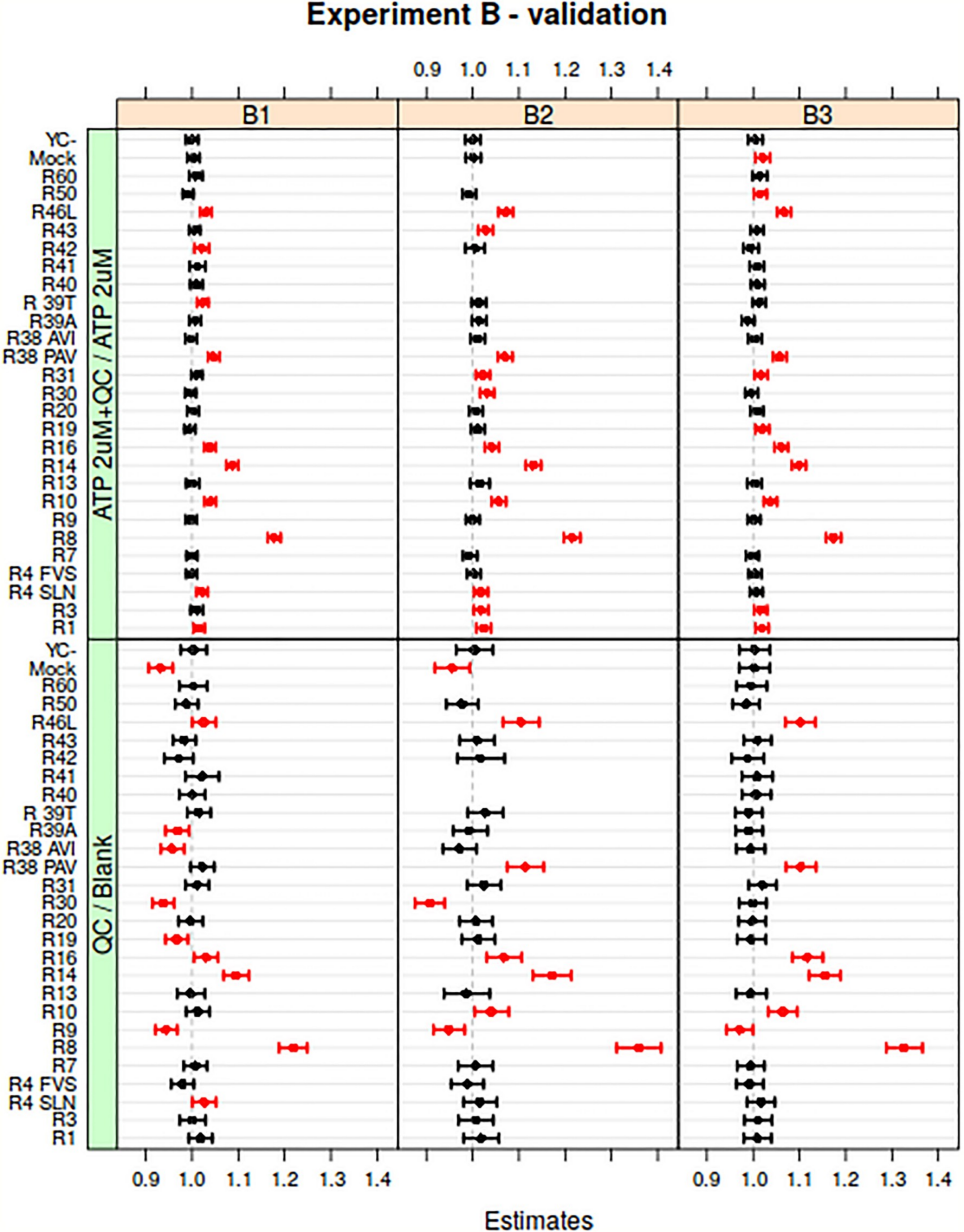
Validation of mixed-model results in the presence of a strong background. The top panels show the estimated treatment-versus-control contrasts comparing spiked and non-spiked ATP injections. Even though the ATP will lead to a strong signal it is possible to pick up the receptors responding to the compounds in the QC mixture mentioned in Table 1. The bottom panels show the results of directly comparing the pure QC mixture injections with the blank, so without the ATP background signal. The top and bottom rows are generally in good agreement, even though the bottom panels show much more variation and wider confidence intervals due to the lower number of replicated injections.

The estimated contrasts and confidence intervals for the three replicated B experiments are shown in the top panels of Fig 6. Even though the ATP leads to a large signal, varying in size depending on the type of the receptor, the analysis is able to identify the effects of the QC mixture on all receptors mentioned in Table 1—six receptors, R8, R10, R14, R16, R38 PAV and R46L seem to stand out in particular. The joint analysis of the ATP-containing injections for the three experiments is shown in S8 Fig. The two control receptors, Mock and YC-, overall have non-significant effects, as expected.

Corresponding results from the model fitted using only the Blank (the reference) and QC injections are shown in the bottom panels in Fig 6. Even though this is supposedly a much easier task since the large ATP background is absent in this comparison, the results are much more variable: the contrast estimates themselves show large variation between repeated experiments, and the estimated confidence intervals are much wider. The main reason for this is that the replication level is lower: the blank was injected only once, and the QC mixture twice. In contrast, there are four non-spiked ATP and three spiked ATP injections. Nevertheless, this clearly shows that the mixed model is able to efficiently pick up signal also in the presence of a large background.

## Discussion

Data from sensors based on live cells often lead to highly variable results. At the cell level, transfection efficiency and cell-cycle differences cause variation in protein expression, leading to variation at the spot level. This can be prominent if relatively few cells make up a signal on a receptor-cell microarray. In addition, in the analysis of complex samples both receptor-specific and generic host-cell responses are often encountered.

Here, these issues have been tackled in several ways. First of all, the design of the slide is important. The high replication of receptors of interest (typically, ten spots are printed for all receptor types) leads to more precise estimates. Perhaps even more importantly, a high replication provides a safeguard against “bad” spots—we are working with live cells so their behaviour is not always predictable. In the preprocessing phase a number of spots showing too little signal are typically eliminated. Since one of the main characteristics of the analysis pipeline proposed in this paper is to get rid of spot effects, we still should have enough spots for each receptor type to do this. For instance, for experiment B1, using only half the number of spots would on average lead to a 7% increase in the width of the confidence intervals and more variability in the contrast estimates. Note that the current array can easily accomodate all our bitter receptors and achieve high replication; it is also possible to simply print larger arrays containing more spots.

Secondly, quality control measures can be taken in the preprocessing, before the statistical analysis: removing spots represented by too few pixels in the raw images has already been mentioned. Sometimes air bubbles prevent spots from being covered by the fluid containing the sample—such spots would be flagged manually and removed from the analysis (this was not the case in the data presented in this paper). Additional quality control steps can easily be integrated if necessary. Thirdly, and the main topic of the current paper, by choosing an appropriate statistical model to describe the behaviour of the system very detailed and quantitative information can be obtained. The mixed models proposed here allow one to draw conclusions about whether or not two samples are different, and if so, what receptors are involved in sensing the differences between the samples. The results are given as tables including estimates and confidence intervals, and can be easily visualized. Of particular importance is the fact that the variation between spots of the same type, an important factor in the overall variability, can be separated from the relevant information by limiting the comparisons to be done within spots. In that sense, the mixed-model approach leads to more precise and more easily interpretable answers. This is true not only for the flow system described here but also in conventional methods using microtiterplates if repeated sample exposure could be achieved. Even the presence of a large generic host-cell response does not prevent the analysis from obtaining correct and precise results.

The current data-analysis set-up is very flexible and can be changed and adapted in several different ways. We can use other response variables, we can use more complicated models, or we can combine the results of the current models in a different way.

### Other response variables

In this paper, the response of a spot is expressed as a ratio of the initial signal and the signal at the top of the peak. Obviously, other measures could have been chosen, such as the difference between the two signal values rather than the ratio. This would correspond to a simple measure of peak height. For the statistical analysis, however, this would lead to heteroscedastic data: high peaks would show much more variability than low peaks, necessitating more complex statistical models. A logarithmic transform of peak heights, which would at least partially alleviate the effects of such heteroscedasticity, is usually not possible since spot responses may be zero or even become lower after sample injection, leading to zero or negative peak heights. Therefore, our default is to use the log-scaled signal ratios described in this paper. Nevertheless, there may be cases where one is explicitly interested in fitting models for peak height. Also other response variables could be envisaged, such as the peak area, the degree of tailing of a peak, or the maximal steepness of the slope. Finally, in experiments where the timing of the response (early or late) is important one could, *e.g*., consider the time to reach the top of the peak, or to the start of the peak, as the response variable.

### More complicated statistical models

The statistical model describing the behaviour of the system can be extended easily—this is one of the most attractive features of the current approach and a defining difference with the usual strategy of simply averaging spots of the same receptor type. One example of a simple extension is the linear term describing the decrease in response over a series of injections employed in the data from Experiment B. In this paper, we have used one slope to describe all spots, but one could actually fit receptor-dependent or even spot-dependent slopes, or fit non-linear slopes such as an exponential decay. One could take into account interactions between subsequent injections—cells may respond differently to a stimulus depending on what happened in the near past. However, more complicated models obviously consume more degrees of freedom (and take more calculation time). The goal is to obtain an adequate description (using whatever definition appropriate) of the behaviour of the system with minimal resources. The set-up used in this paper takes a couple of seconds at most using simple every-day hardware, so is eminently usable in practice.

### More complicated comparisons

We have shown that it is possible to obtain very good results in comparing spiked samples with non-spiked samples: the proposed methodology is able to eliminate the common background signal (triggered by ATP) from the receptor-specific signals, even though the background is the largest component by far. In more general experiments, where the differences between the injection types are more complicated than the simple addition of a spike mixture, it may not be so easy to remove host-cell responses: in pathological cases host-cell responses may completely cancel out receptor-specific responses. It is clear from the results in this paper that each receptor spot has a different host-cell response, and it is not correct to simply assume that the Mock spots provide a good estimate. We are currently exploring ways to disentangle the effects in a general way.

## Conclusion

In the field of receptomics, microfluidic receptor-cell arrays are a valuable tool in investigating human responses to food and in trying to understand the relation between chemical composition and taste in food stuffs [6, 21]. We have shown that careful experimental design, data processing and statistical analysis can lead to highly informative results, opening the way to many diverse applications. One particularly interesting possibility is to use these cell arrays as extensions of taste panels for prescreening: in principle, many quantitative comparisons can be made in a rapid and cost-effective way. This receptomics tool can be further extended to other receptors of the GPCR gene family and ion channels, both of humans and other organisms.

Because of the complexity of the underlying biological processes, statistical analysis is non-trivial. In this paper, a consistent and robust strategy has been devised, validated and implemented in the form of R scripts to achieve maximum flexibility and transferability. On top of these scripts, a software package has been built, focusing on the models described in this paper, allowing also non-specialists to access the power of the mixed-model analyses, thus greatly reducing the time needed to analyse these complex data sets, and ensuring reproducibility of the results.

## Supporting information

S1 Table Overview of bitter raste receptor genes used in the bitter-receptor array. Polymorphism variants are indicated with the aminoacid number of the receptor protein and the variable amino-acid letter code. In brackets the printed polymorphism is shown. The rightmost column contains the number of replications on the array.
(PDF)

S1 Code scripts.R: File containing R scripts for reproducing the results (including figures) in this paper. (R)

S1 Data ExperimentA.csv: File containing data for the three experiments of type A. (CSV)

S2 Data ExperimentB.csv: File containing data for the three experiments of type B. (CSV)

S1 Fig Data processing: From CFP and YFP signals to spot signals. The top panel shows the raw CFP and YFP signals for spot 21 from the first type-B experiment (a spot of receptor type R8). These raw signals are smoothed and interpolated to obtain values for exactly the same time points (shown in the middle panel). The final spot signal is calculated as YFP/CFP, and is shown in the bottom panel.
(TIFF)

S2 Fig Definition of the magnitude of the spot response to an injection. The plots depict the last three injections of the data also shown in S1 Fig. The magnitude of the spot response is the ratio of the extreme point within a time window (here 30 cycles, indicated by the gray vertical lines), and the starting value, the average of the first three points.
(TIFF)

S3 Fig PCA loading plots for the three replicated experiments, type A. Spots of types R8 and R14 are highlighted to show the variability between spots of the same receptor type. For clarity, the largest loadings are shown with arrows, smaller ones are shown with dots only.
(TIFF)

S4 Fig Estimated treatment-versus-control contrasts for individual spotmixes in experiment A1. Each panel contains the result of one receptor type; dilutions are given at the *y* axis, with stronger dilutions towards the bottom. Significant contrasts, not containing the value of one in the confidence interval, are indicated in red. The blank injection serves as the reference.
(TIFF)

S5 Fig Estimated treatment-versus-control contrasts for individual spotmixes in experiment A2. For explanation, see legend of S4 Fig.
(TIFF)

S6 Fig Estimated treatment-versus-control contrasts for individual spotmixes in experiment A3. For explanation, see legend of S4 Fig.
(TIFF)

S7 Fig PCA loading plots for the three replicated experiments, type B. Spots of types R8 and R14 are highlighted to show the variability between spots of the same receptor type. For clarity, the largest loadings are shown with arrows, smaller ones are shown with dots only.
(TIFF)

S8 Fig Joint analysis of type-B experiments. For explanation, see the caption of Fig 3. The reference level is given by the injection type ATP 2uM.
(TIFF)
