# Supplementary material for: Statistical models discriminating between complex samples measured with microfluidic receptor-cell arrays
Source: PLoS One. 2019 Apr 8;14(4):e0214878. doi: 10.1371/journal.pone.0214878 (PMC6453450; doi:10.1371/journal.pone.0214878)
Supplement: S1 Table — Polymorphism variants are indicated with the aminoacid number of the receptor protein and the variable amino-acid letter code. In brackets the printed polymorphism is shown. The rightmost column contains the number of replications on the array. (PDF) [file pone.0214878.s001.pdf]

Table 1: **Overview of bitter raste receptor genes used in the bitter-receptor array.** Polymorphism variants are indicated with the aminoacid number of the receptor protein and the variable amino-acid letter code. In brackets the printed polymorphism is shown. The rightmost column contains the number of replications on the array.

Receptor number	Name	Polymorphisms	#
1	Tas2R1		10
2	Tas2R3		10
3	Tas2R4	F7S, V96L, S171N (FVS)	10
4	Tas2R4	F7S, V96L, S171N (SLN)	10
5	Tas2R5		10
6	Tas2R7		10
7	Tas2R8		10
8	Tas2R9		10
9	Tas2R10		10
10	Tas2R13		9
11	Tas2R14		10
12	Tas2R16		10
13	Tas2R19		10
14	Tas2R20		10
15	Tas2R30		10
16	Tas2R31		10
17	Tas2R38	A49P, V262A, I296V (PAV)	10
18	Tas2R38	A49P, V262A, I296V (AVI)	10
19	Tas2R39	A301T (A)	10
20	Tas2R39	A301T (T)	10
21	Tas2R40		10
22	Tas2R41		10
23	Tas2R42		10
24	Tas2R43		10
25	Tas2R46		10
26	Tas2R50		10
27	Tas2R60		10
28	Mock		8
29	YC-		8
