# Supplementary figures and images for: Statistical models discriminating between complex samples measured with microfluidic receptor-cell arrays
Source: PLoS One. 2019 Apr 8;14(4):e0214878. doi: 10.1371/journal.pone.0214878 (PMC6453450; doi:10.1371/journal.pone.0214878)

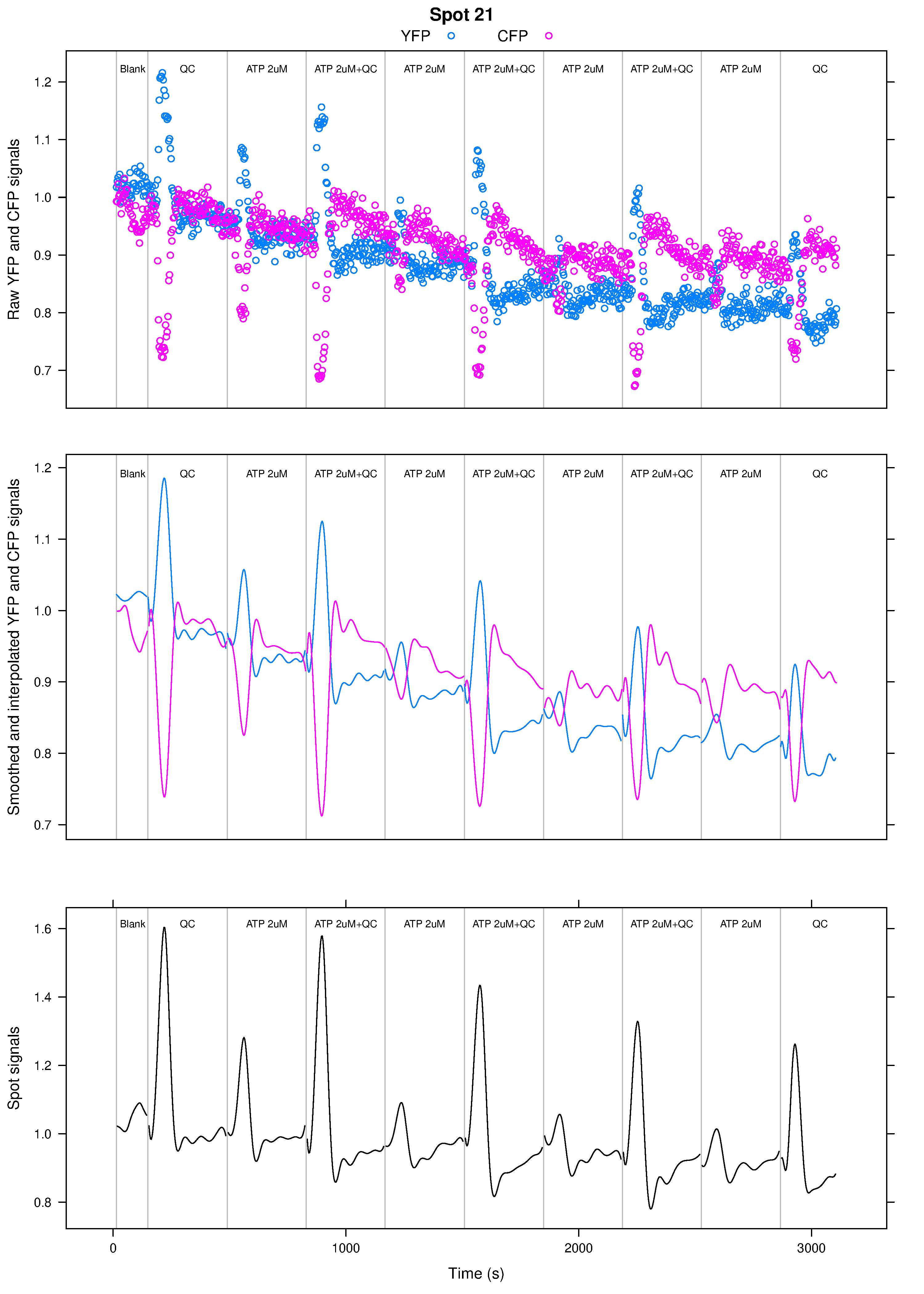

Supplement: S1 Fig — The top panel shows the raw CFP and YFP signals for spot 21 from the first type-B experiment (a spot of receptor type R8). These raw signals are smoothed and interpolated to obtain values for exactly the same time points (shown in the middle panel). The final spot signal is calculated as YFP/CFP, and is shown in the bottom panel. (TIFF) [file pone.0214878.s005.tiff]

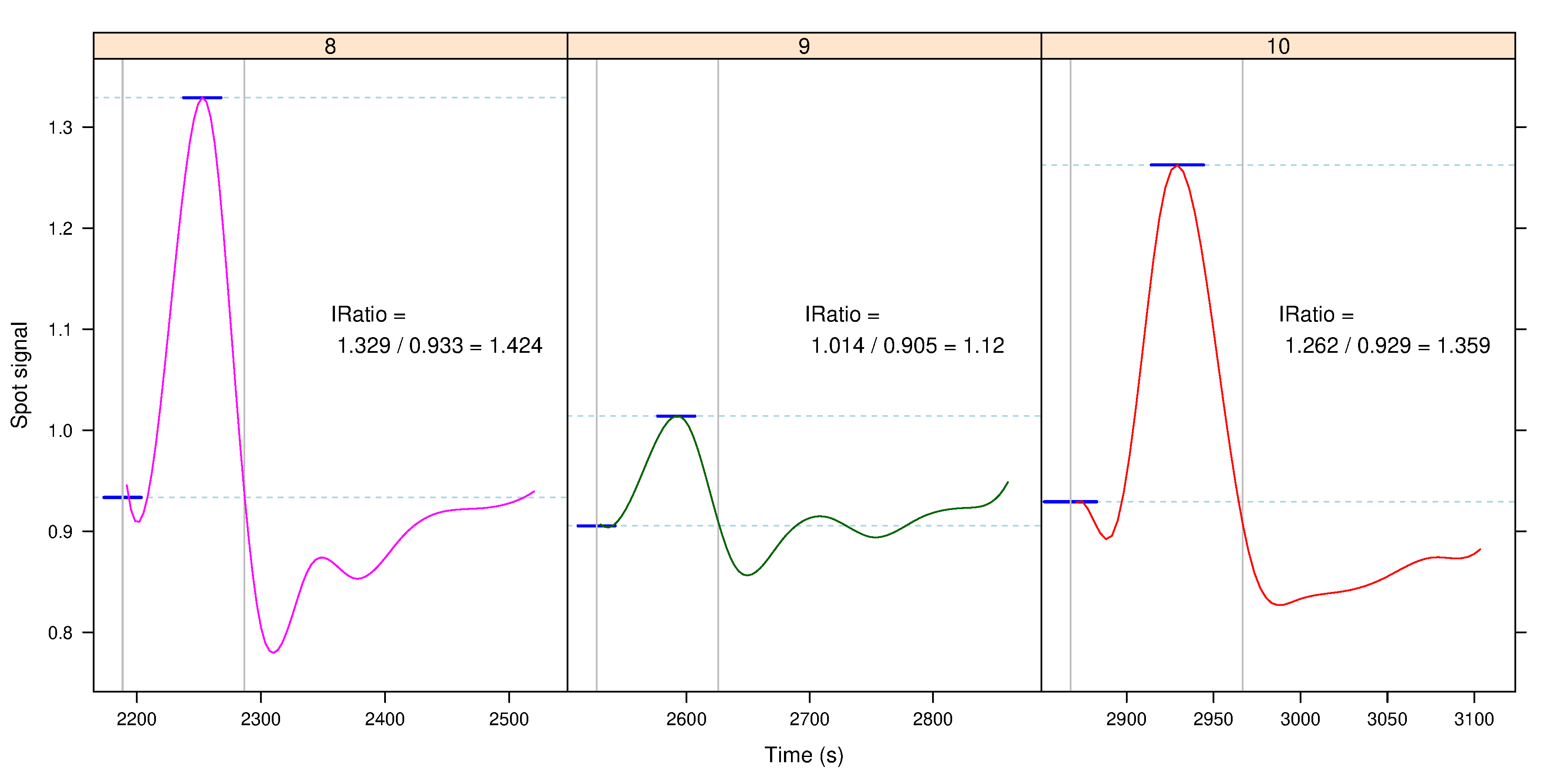

Supplement: S2 Fig — The plots depict the last three injections of the data also shown in S1 Fig. The magnitude of the spot response is the ratio of the extreme point within a time window (here 30 cycles, indicated by the gray vertical lines), and the starting value, the average of the first three points. (TIFF) [file pone.0214878.s006.tiff]

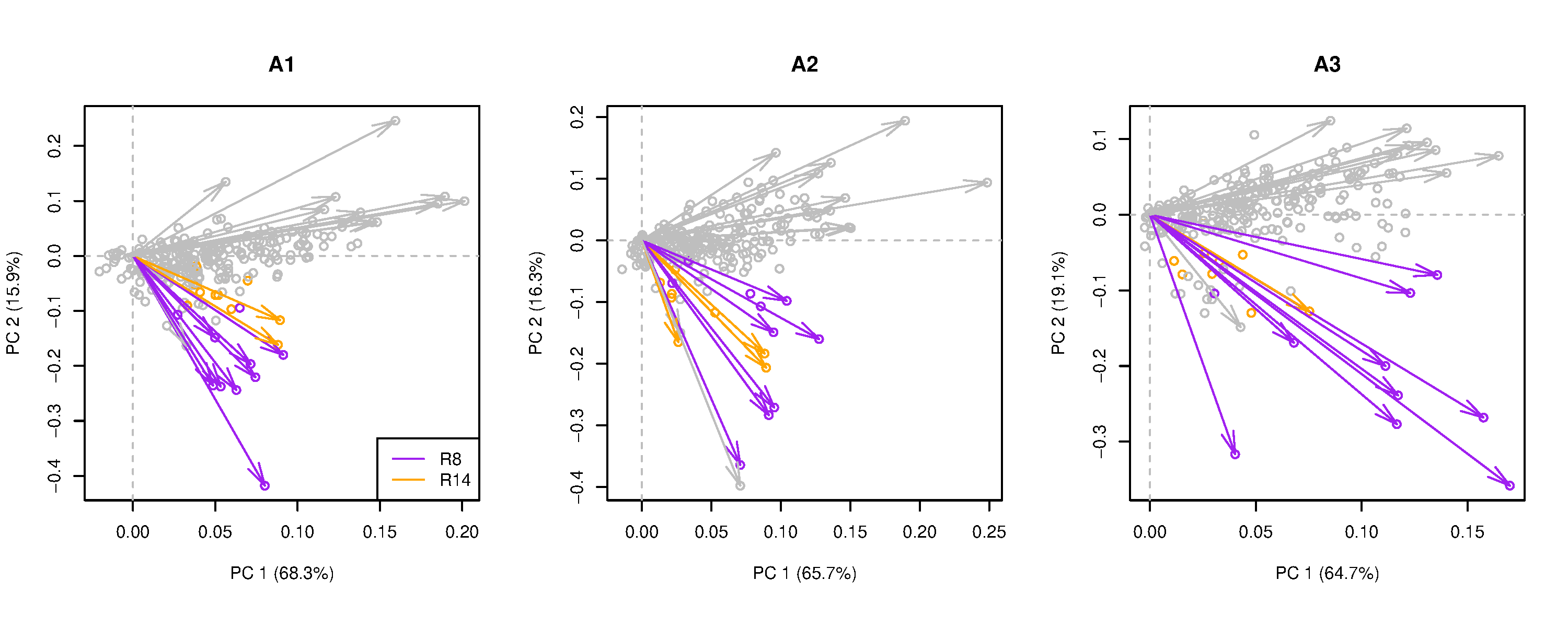

Supplement: S3 Fig — Spots of types R8 and R14 are highlighted to show the variability between spots of the same receptor type. For clarity, the largest loadings are shown with arrows, smaller ones are shown with dots only. (TIFF) [file pone.0214878.s007.tiff]

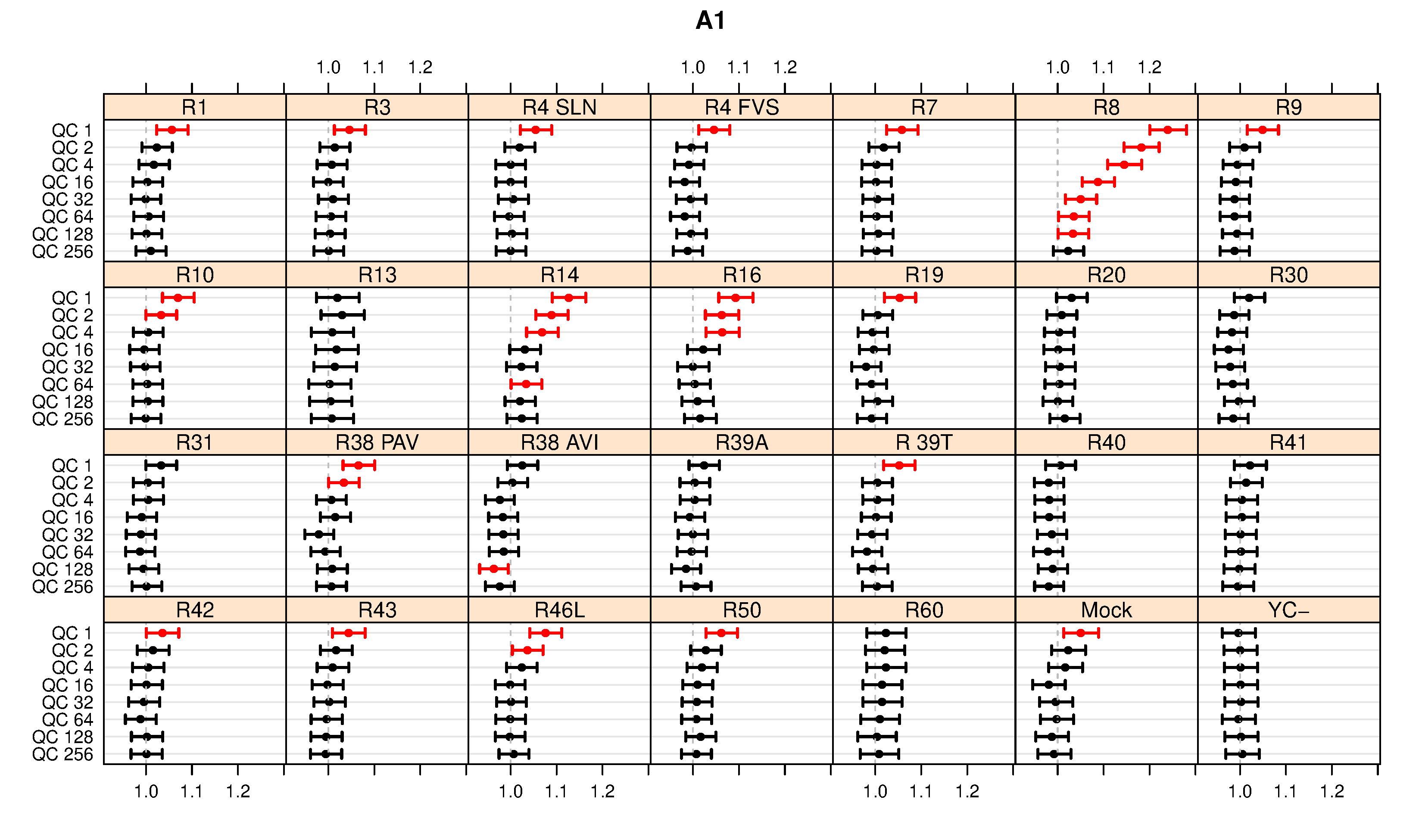

Supplement: S4 Fig — Each panel contains the result of one receptor type; dilutions are given at the y axis, with stronger dilutions towards the bottom. Significant contrasts, not containing the value of one in the confidence interval, are indicated in red. The blank injection serves as the reference. (TIFF) [file pone.0214878.s008.tiff]

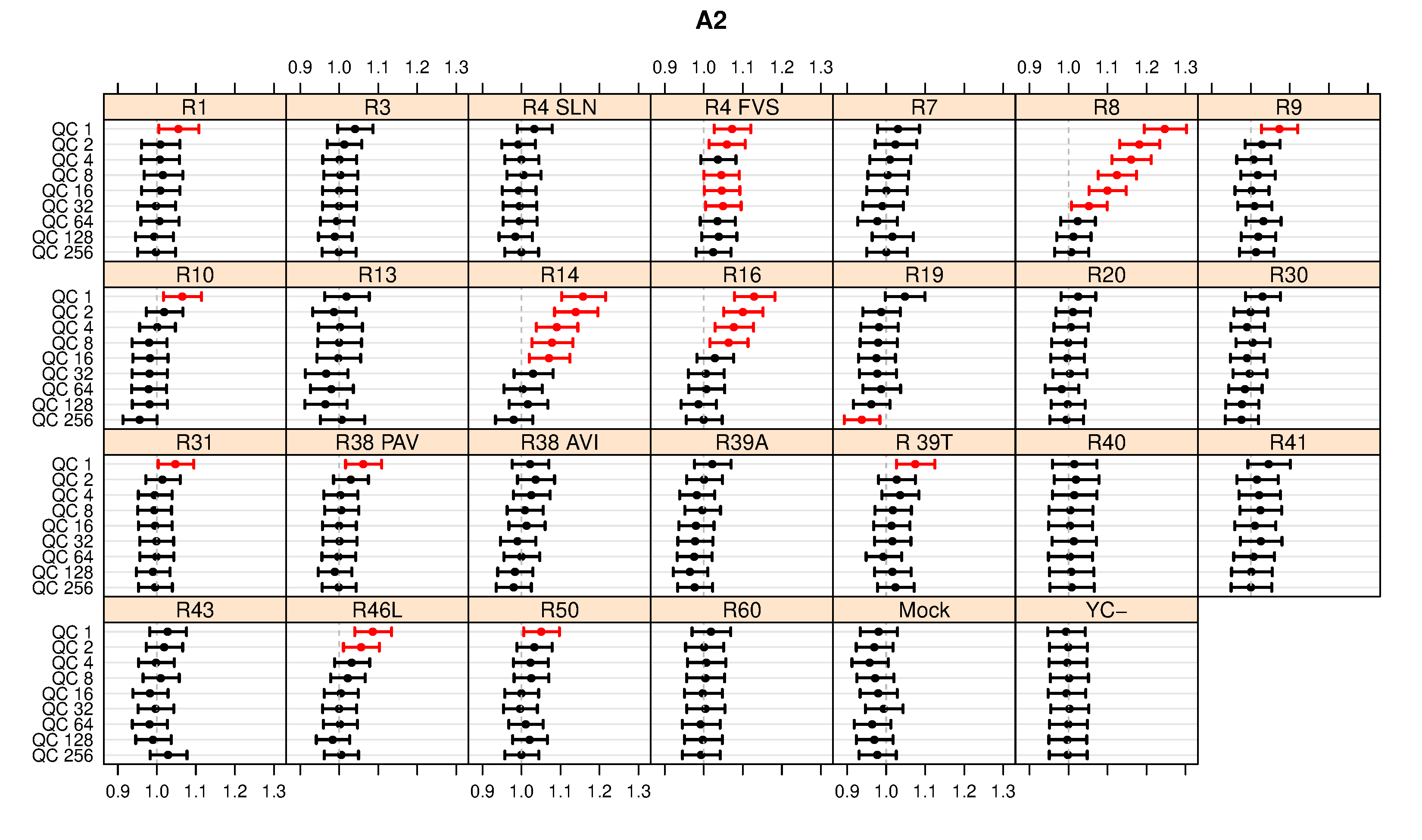

Supplement: S5 Fig — For explanation, see legend of S4 Fig. (TIFF) [file pone.0214878.s009.tiff]

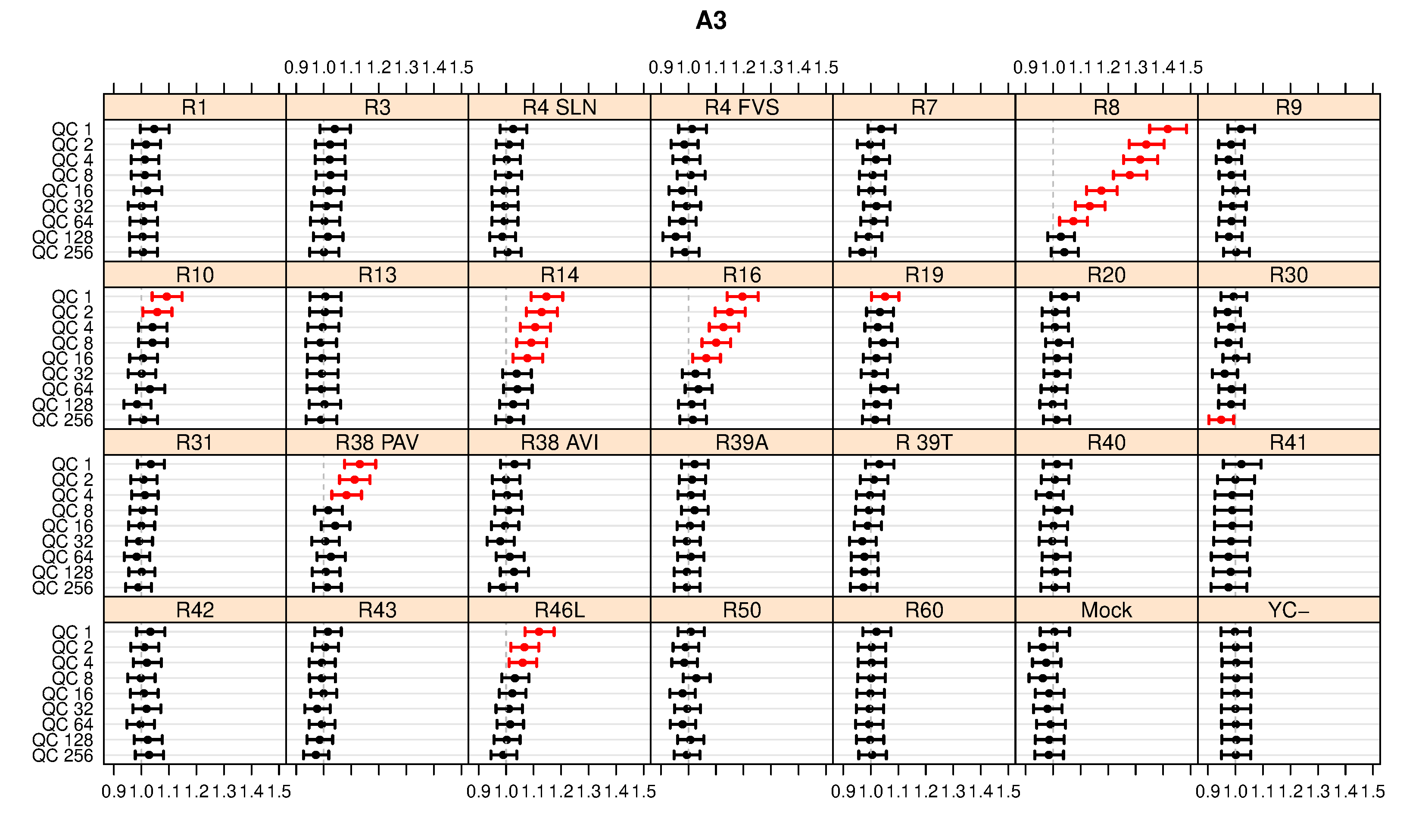

Supplement: S6 Fig — For explanation, see legend of S4 Fig. (TIFF) [file pone.0214878.s010.tiff]

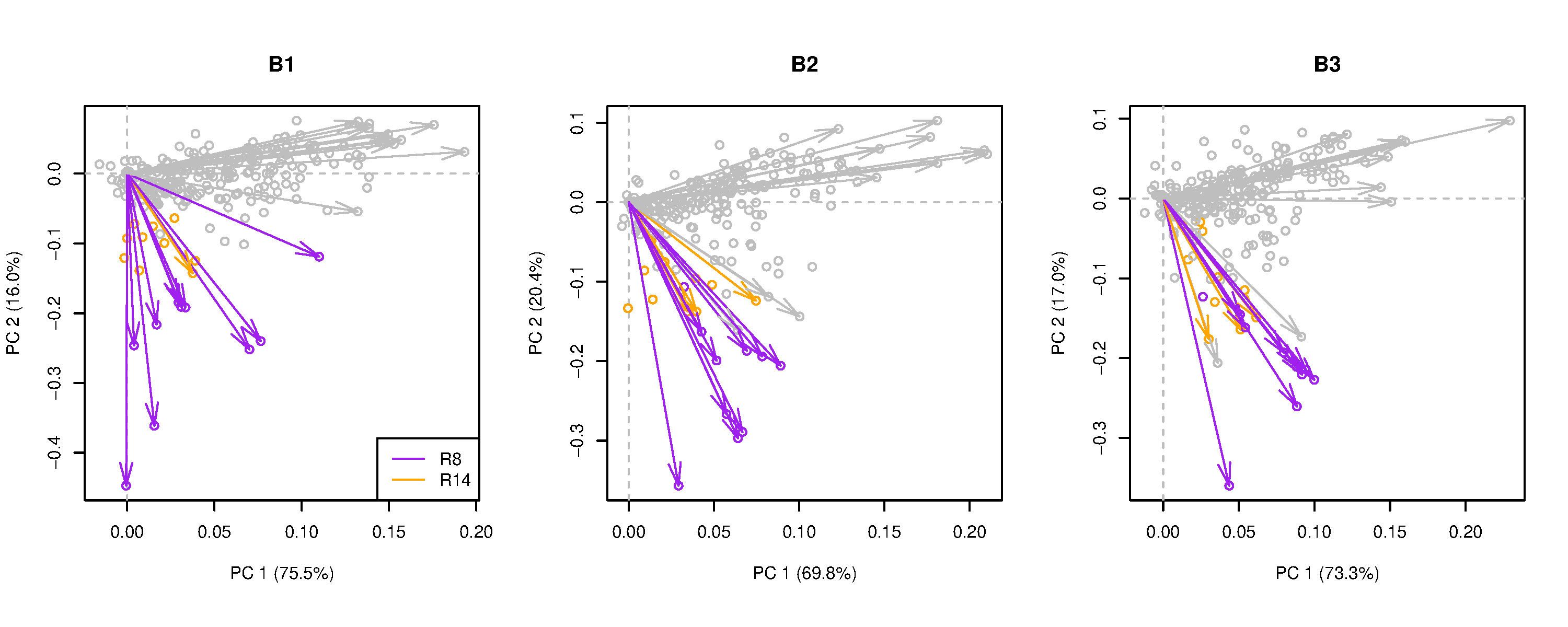

Supplement: S7 Fig — Spots of types R8 and R14 are highlighted to show the variability between spots of the same receptor type. For clarity, the largest loadings are shown with arrows, smaller ones are shown with dots only. (TIFF) [file pone.0214878.s011.tiff]

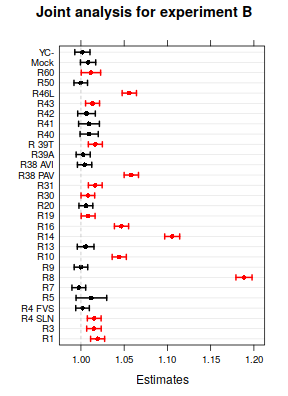

Supplement: S8 Fig — For explanation, see the caption of Fig 3. The reference level is given by the injection type ATP 2uM. (TIFF) [file pone.0214878.s012.tiff]
